# Thinking outside the cranium

**Published:** 2014-07-04

**Authors:** Anahita Sadeghi, Behrouz Navabakhsh, Leila Aghaghazvini

**Affiliations:** 1 Department of Internal Medicine, School of Medicine, Shariati Hospital, Tehran University of Medical Sciences, Tehran, Iran; 2 Department of Radiology, School of Medicine, Shariati Hospital, Tehran University of Medical Sciences, Tehran, Iran

**Keywords:** Cerebral Toxoplasmosis, Human Immunodeficiency Virus, Eccentric Target Sign, Magnetic Resonance Imaging

## Introduction

Cerebral toxoplasmosis is a leading cause of central nervous system (CNS) lesions in severely immunocompromised human immunodeficiency virus+ (HIV+) patients who are seropositive for the parasite and have not received effective chemoprophylaxis. A 48-year-old HIV+ woman presented to our emergency room with seizure and decreased level of consciousness. She had developed fever, productive cough, and malaise 1 week prior to her current admission. Multifocal sensory and motor deficits in her extremities were revealed on neurologic examination. The brain imaging of our patient showed a distinctive ring-enhancing lesion that narrowed our differential diagnosis considerably.

The patient was admitted on January 20, 2009 with decreased level of consciousness, urinary incontinence, upward eye deviation, right arm tonic flexion and right leg clonic jerks while the left extremities were tonically extended. Her seizure was controlled with intravenous diazepam and phenytoin, and she was fully conscious for history taking 5 h after presentation.

The patient had a positive history for HIV infection and reported prolonged close contact with a known tuberculosis (TB) case 2 years ago. She had developed fever, productive cough and malaise 1 week prior to her current admission; CD4 count of 18 cells/μL, skin purified protein derivative reactivity of 5 mm and a negative sputum smear for acid-fast bacilli had been reported at the time waiting for culture results. She also complained of headaches and weight loss during the past month. Oral co-amoxiclav and co-trimoxazole were administered for her recent symptoms. For her HIV, she had received IMOD intermittently (for a total of 6 months) during the year prior to admission – Setarud (IMOD) is a herbal medicine prepared from a mixture of extracts of *Rosa canina*, *Urtica dioica*, and *Tanacetumv ulgare* in addition to selenium, flavonoids, and carotenes. Ten years ago, after a bout of jaundice, she had been diagnosed with acute hepatitis B. The resolution of which was documented with hepatitis B surface antigen clearance and hepatitis B surface antibody seroconversion at the time.

**Figure 1:**
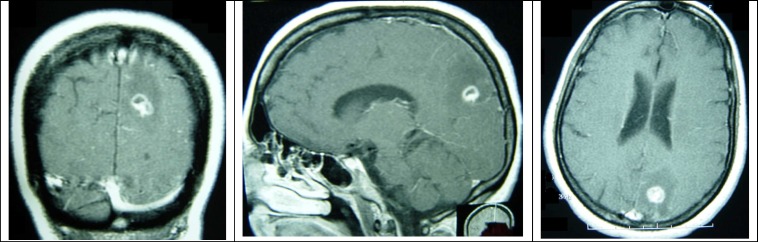
T_1_-weighted magnetic resonance imaging with gadolinium enhancement shows a single 1.5 cm targetoid lesion with ring enhancement and parenchymal vasogenic edema surrounding the lesion in the left posterior parietal lobe

Neurologic examination revealed multifocal sensory and motor deficits involving her left ulnar nerve distribution, right median nerve distribution, proximal and distal right lower extremity weakness and atrophy, and symmetric sensory deficits in both feet. The patient’s lab data during the past year revealed a steady decline in CD4 counts from 361 cells/μL in August 2007 and variable peripheral eosinophilia. The patient’s lab results during her admission are shown in table 1. Brain imaging showed a single ring-enhancing lesion in the left posterior parietal lobe (Figure 1).

In developing countries, the differential diagnosis for a severely immunocompromised HIV+ patient with a solitary ring-enhancing brain lesion should include primary CNS lymphoma (PCNSL), TB, toxoplasmosis, neurocysticercosis, and much less commonly infections caused by *Staphylococcus*, *Streptococcus*, *Salmonella*, *Aspergillus*, *Nocardia*, *Rhodococcus*, *Listeria*, *Cryptococcus*, *Treponemapallidum*, and cytomegalovirus (CMV).

The typical magnetic resonance finding in toxoplasmic encephalitis (TE) is that of multiple ring-enhancing lesions with a predilection for basal ganglia; solitary lesions are reported in approximately 30% of cases.^1^ Single and multiple lesions are equally common in patients with PCNSL and because of the inadequacy of neuroradiological features to distinguish between TE and PCNSL, a high suspicion for the latter should be maintained throughout the follow-up of a presumed TE patient with a solitary CNS lesion – especially for lesions that are larger than 4 cm. The “eccentric target sign” in post-contrast T_1_-weighted magnetic resonance imaging (MRI) has been proposed as a highly characteristic feature found in 30% of biopsy proven cerebral toxoplasmosis cases.^2^ This imaging feature is more commonly found in cortical-based toxoplasmosis abscesses organizing around a cerebral sulcus with the eccentric core representing the inflamed cerebral vessels traversing that sulcus.^3^ Neurocysticercosis can produce a similar ring-enhancing lesion with an eccentric hyperdense nodule representing the scolex of the live tapeworm, but the parasite cysts in this stage of development can usually be differentiated from an abscess by the absence of diffusion restriction within the cyst in diffusion-weighted imaging and minimal parenchymal edema surrounding the lesion;^4^ in addition, *Taenia solium* infections are extremely rare in Iran.

Empiric treatment for TE as the most probable cause of the patient’s CNS lesion was began with pyrimethamine, sulfadiazine, and leucovorin. Highly active antiretroviral therapy for her HIV infection and isoniazid for her TB infection were also initiated as well as co-trimoxazole and azithromycin for *Pneumocystis jiroveci* pneumonia and *Mycobacterium avium* complex prophylaxis. HIV infection by itself was identified as the probable cause of the patient’s eosinophilia.^5^

**Table 1:** Laboratory data

**Variable**	**Reference range**	**January 22** ^nd^ **, 2009**
White cell count (per mm^3^)	4000-11,000	6800
Differential count (%)		
Neutrophils	40-70	46
Lymphocytes	22-44	43
Monocytes	2-10	2
Eosinophils	0-8	9.8
Basophils	0-3	0
Hemoglobin (g/dL)	13-16 (women)	9.8
Platelet count (per mm^3^)	150,000-450,000	318,000
ESR (mm/h)	0-15	45
CRP (mg/L)	< 10	160
ALT (IU/L)	Up to 38	10
AST (IU/L)	Up to 40	12
ALP (IU/L)	80-306	105
INR	1-1.2	1.1
PTT (s)	25-35	22
Urea (mg/dL)	7-21	27
Creatinine (mg/dL)	0.7-1.4	1.1
LDH (IU/L)	Up to 480	317
Blood sugar (mg/dL)	Up to 140	110
Calcium (mg/dL)	8.5-10.5	9.6
Phosphorus (mg/dL)	2.5-5	3.1
Sodium (meql/L)	135-145	139
Potassium (meq/L)	3.5-5	4.7
VDRL		Negative
Toxoplasma IgG		Positive
Toxoplasma IgM		Negative
CMV IgG		Positive
Anti-HBs		Negative
HBsAg		Negative
Anti-HCV		Negative

ESR: Erythrocyte sedimentation rate; CRP: C-reactive protein; ALT: Alanine transaminase; AST: Aspartate aminotransferase; ALP: Alkaline phosphatase; INR: International normalized ratio; PTT: Partial thromboplastin time; LDH: Lactate dehydrogenase; VDRL: Venereal Disease Research Laboratory; IgG: Immunoglobulin G; IgM: Immunoglobulin M; CMV: Cytomegalovirus; Anti-HBs: Hepatitis B surface antibody; HBsAg: Hepatitis B surface antigen; HCV: Hepatitis C virus

Despite being rare, CMV infection can present as ring-enhancing lesions in severely immunocompro-mised HIV patients and because of the presence of mononeuropathy multiplex in this patient was considered a possibility. Since PCNSL, TB, and CMV could not be definitely ruled out, a follow-up MRI after 2 weeks of empirical therapy was planned. Our patient responded favorably to the initial empiric therapy thus consolidating a diagnosis of cerebral toxoplasmosis.

## Conclusion

The most probable diagnosis of cerebral lesion in HIV positive patients is cerebral toxoplasmosis and in resource poor settings for cerebral biopsy, the patients can be treated according to radiographic and laboratory findings.

## Notes

**How to cite this article:** Sadeghi A, Navabakhsh B, Aghaghazvini L. Thinking outside the cranium. Iran J Neurol 2014; 13(3): 188-90.
